# Colonization and spatiotemporal distribution of bruchid pests in lentil and faba bean fields

**DOI:** 10.1002/ps.70679

**Published:** 2026-02-23

**Authors:** Anastasia Chery‐Lagrange, Antoine Gardarin, Yann Tricault, Mélissandre Gabet, Jean‐David Chapelin‐Viscardi, Cayetano Herrera, Anne‐Sophie Voisin, Laurent Bedoussac

**Affiliations:** ^1^ Univ Toulouse Toulouse INP, PURPAN, INRAE, AGIR Castanet‐Tolosan France; ^2^ Agronomie, Université Paris‐Saclay AgroParisTech, INRAE Palaiseau France; ^3^ IGEPP, INRAE, Institut Agro Univ Rennes Angers France; ^4^ Laboratoire d’Eco‐Entomologie Orléans France; ^5^ Agroecologie, Univ Bourgogne Univ‐Franche‐Comté, INRAE Dijon France; ^6^ Univ Toulouse Toulouse INP, ENSFEA, PURPAN, INRAE, AGIR Castanet‐Tolosan France

**Keywords:** Pest monitoring, population dynamics, grain legume, pest management, agroecology, phenology

## Abstract

**BACKGROUND:**

Lentils (*Lens culinaris* Medikus, 1787) and faba beans (*Vicia faba* Linnaeus, 1753) are important crops in France facing threats from *Bruchus* spp*.* We analyzed 59 lentil and 45 faba bean fields across four French regions over three growing seasons (2019–2020 to 2021–2022). We investigated the diversity, colonization patterns and spatiotemporal distribution of bruchids at different crop phenological stages and distances from field edges.

**RESULTS:**

*Bruchus rufimanus* Boheman, 1833 and *Bruchus signaticornis* Gyllenhal, 1833 were the only species emerging from faba beans (97.8%) and lentils (99.5%), respectively. *B. rufimanus* colonization was concentrated during pod development, maintaining a balanced male–female ratio throughout. *B. signaticornis* exhibited a colonization period of ≈1 month, with a gradual increase in female proportion over time. The spatial distribution of bruchids and damage were relatively uniform within fields, indicating strong dispersal capabilities. A significant positive correlation, with a high degree of dispersion, was identified between female abundance and bruchid‐damaged grains.

**CONCLUSION:**

We confirmed that *B. rufimanus* and *B. signaticornis* were the only species damaging faba beans and lentils in France, respectively. The homogeneous spatial distribution suggests a strong dispersal ability of bruchids. The high degree of dispersion in the relationship between female abundance and bruchid‐damaged grains highlights the importance of regulatory factors influencing larval and egg survival. These results, together with the presence of *B. signaticornis* in faba beans, emphasize the need for species‐specific, phenology‐based and spatially informed integrated pest management strategies, to mitigate the impact of bruchids and reduce reliance on chemical in their control. © 2026 The Author(s). *Pest Management Science* published by John Wiley & Sons Ltd on behalf of Society of Chemical Industry.

## Graphical abstract

This study of 104 fields identifies *Bruchus signaticornis* in lentils and *B. rufimanus* in faba beans as the sole grain‐damaging species. Their uniform distribution within fields and the strong effect of regulatory factors on larvae and egg survival emphasize the need for species‐specific, phenology‐based and spatially informed integrated pest management strategies.

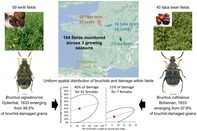

## 1 INTRODUCTION

Climate change and biodiversity loss call for the development of more sustainable and environmentally‐friendly cropping systems, as part of an agroecological and food transition.^1^ In this context, grain legumes offer two major ecological and nutritional advantages. First, their ability to fix atmospheric nitrogen allows for a substantial reduction in the use of mineral fertilizers, whose production contributes significantly to greenhouse gas emissions, and can thus help to combat climate change.^2^ Second, grain legumes are rich in both protein and carbohydrate, making them high‐quality foodstuffs for human consumption and the transition to a more nutritious diet.^3^

Among the various grain legumes, lentils (*Lens culinaris* Medikus, 1787) are the most popular in France for human food and rank eighth worldwide, with an average global annual production of 6 million t between 2014 and 2019.^4^, ^5^ Faba bean (*Vicia faba* Linnaeus, 1753) is also one of the most widely consumed grain legumes by humans in the world, but in France it is mostly grown for animal feed.^6^ In Europe, grain legumes are cultivated on <2% of the total arable land area, despite their ecological, nutritional and agronomic benefits.^7^ This is mainly a consequence of their susceptibility to numerous biotic stresses, which limit their productivity and contribute to unstable yields, both in organic farming and in conventional systems using plant protection products. Biotic stressors include diseases such as *Peronospora lentis*, *Botrytis fabae* or *Uromyces viciae‐fabae*, as well as insect pests such as *Sitona lineatus*, *Bruchus* spp. and *Aphis craccivora*.^8^

Among the various insect pests affecting lentils and faba beans, bruchid beetles of the genus *Bruchus* (Coleoptera: Chrysomelidae: Bruchinae) pose a real challenge to the wider adoption of these crops by farmers. Bruchids show a marked trophic specialization, with *B. signaticornis* Gyllenhal, 1833 on lentil and *B. rufimanus* Boheman, 1833 considered as the main pest species, in France and Europe.^9^, ^10^, ^11^ Recent literature suggests that many earlier works contained species identification errors, or failed to distinguish clearly between larval feeding within grains and adult feeding on plants and may therefore lead to an overestimation of the number of species capable of successfully completing their larval development on faba bean and lentil.^12^, ^13^, ^14^, ^15^, ^16^, ^17^, ^18^ To date, 13 species are listed in the literature as being capable of successfully completing their larval development on lentils (*B. atomarius*, *B. brachialis*, *B. ervi*, *B. lentis*, *B. griseomaculatus*, *B. loti*, *B. luteicornis*, *B. rufimanus*, *B. rufipes*, *B. signaticornis*, *B. tristiculus*, *B. tristis* and *B. ulicis*) and seven species on faba beans (*B. affinis*, *B. atomarius*, *B. dentipes*, *B. pisorum*, *B. rufimanus*, *B. rufipes* and *B. tristis*).^14^, ^15^, ^16^, ^17^, ^18^

Bruchid activity is closely related to host crop phenology.^19^ Flying adults leave semi‐natural habitats or cultivated areas and colonize grain legume crop fields during spring where they reproduce.^9^, ^12^, ^20^ In Europe, bruchids are univoltine species and the adults are capable of feeding on extrafloral nectar, floral nectar and pollen from a variety of cultivated and spontaneous Fabaceae plants. For example, *B. pisorum* is commonly associated with *Pisum sativum*; *B. rufipes* with *Vicia monantha*, *V. sativa* and *V. villosa*; *B. signaticornis* with *Lathyrus annuus* and *Vicia incana*; and *B. rufimanus* with *Lathyrus venetus*, *Vicia bithynica*, *V. faba*, *V. hybrida*, *V. lutea*, *V. narbonensis*, *V. pannonica* and *V. villosa*.^14^ Previous observations in Algeria and in France, mentioned a population peak of *B. rufimanus* between full flowering and the onset of pod formation based on visual observations and sweep net sampling.^19^, ^21^, ^22^ Likewise, in Great Britain, the use of traps containing semiochemical attractants suggested low initial captures of *B. rufimanus* before and during flowering, followed by a massive capture after flowering.^11^ After mating, females lay their eggs on developing pods, and then the larvae that hatch penetrate the pods and develop in the growing grains.^23^ Depending on environmental conditions, larvae may follow one of two phenological strategies: (i) they either complete development and emerge as adults in late summer to overwinter in the surrounding landscape (‘early emergence’), or (ii) remain in the grains for several months until emergence in silos or from sowed seeds the following spring (‘late emergence’).^9^, ^10^, ^24^, ^25^ Infestation rate can be extremely high, reaching ≤70% in both faba bean and lentil.^26^, ^27^, ^28^, ^29^, ^30^, ^31^ In small‐seeded species such as lentil, bruchid larvae consume almost the entire grain. In larger‐seeded species such as faba bean, the relative loss of dry weight (DW) is lower, from 5.0% to 10.0% of seed DW, but the damage causes significant commercial depreciation, especially for grains intended for human consumption.^11^, ^32^ Bruchid infestations also lead to a significant reduction in germination potential.^26^, ^29^, ^33^, ^34^

Conventional management of bruchids, both in the field and during post‐harvest storage, is mainly based on the use of synthetic insecticides.^31^, ^35^ However, these chemicals promote the development of resistance and pose risks to soil health, water quality, biodiversity and human health.^36^, ^37^, ^38^, ^39^ As a result, some of the most effective insecticides, such as endosulfan, a highly toxic organochlorine, have been banned in many countries, particularly in Europe (Endosulfan has been banned in France in 2007).^40^ Innovation in bruchid management therefore focuses on agroecological methods. These include resistant cultivars, chemical‐free storage control methods, attractant traps with kairomones or pheromones for massing trapping, natural repellents with low environmental risk, species mixtures to disrupt host plant detection or even staggering sowing dates to de‐synchronize the bruchid life cycle and crop phenology.^9^, ^23^, ^30^, ^31^, ^41^, ^42^, ^43^, ^44^

All of these options are individually much less effective than synthetic pesticides and must therefore be combined in a systemic approach. Given that they often involve interactions with adult bruchid behaviour, a thorough understanding of their ecology appears to be an essential prerequisite for developing operational agroecological strategies. Studies on bruchid ecology date back to the middle of the last century or concern geographical areas other than France, in particular North Africa.^12^, ^21^, ^22^, ^45^ Recent work has shed new light on *B. signaticornis* on lentil and *B. rufimanus* on faba bean, yet significant gaps in knowledge remain.^10^, ^19^, ^20^ In particular, little is known about the phenology of crop colonization by bruchids depending on crop stage, the spatial distribution of bruchids in cultivated fields throughout the cropping season, the relationship between adult bruchid abundance and the percentage of bruchid‐damaged grains. Although the general life cycle of bruchid species is well‐studied, few studies quantified their population dynamics across growing seasons and among years, or how climatic conditions modulate emergence and colonization.^46^ For example, seasonal abundance of *B. rufimanus* is regulated by climatic conditions and linked to several variables.^47^ Finally, many previous studies have focused on a single bruchid species or a single host crop, which limits the possibility of drawing generic conclusions with notably a view to design integrated pest management strategies against this particular pest of grain legumes.^9^, ^10^, ^11^, ^17^, ^19^, ^20^, ^21^, ^22^, ^24^, ^25^, ^26^, ^27^, ^28^, ^29^, ^30^, ^31^, ^32^, ^33^, ^34^, ^35^, ^41^, ^42^, ^45^, ^47^

The aim of this multiregion and multiyear study is, first, to provide robust and field‐based validation for recent taxonomic revisions under French agroecological conditions.^14^, ^15^, ^16^, ^17^, ^18^ These revisions suggest species identification errors in many earlier works, or failing to distinguish clearly between larval feeding within grains and adult feeding on plants.^14^, ^15^, ^16^, ^17^, ^18^ For this purpose, we analyze the identity of: (i) the bruchid species found as adults in faba bean and lentil crops in France, and (ii) the species responsible for grain damage. Secondly, this work also aims to fill certain knowledge gaps in order to contribute to the development of integrated pest management strategies by analyzing: (i) the spatial and temporal distribution of adult bruchids in cultivated fields, and (ii) the relationship between the percentage of bruchid‐damaged grains and bruchid populations in fields.

## 2 MATERIALS AND METHODS

### 2.1 Location and selection of fields

**Figure 1:**
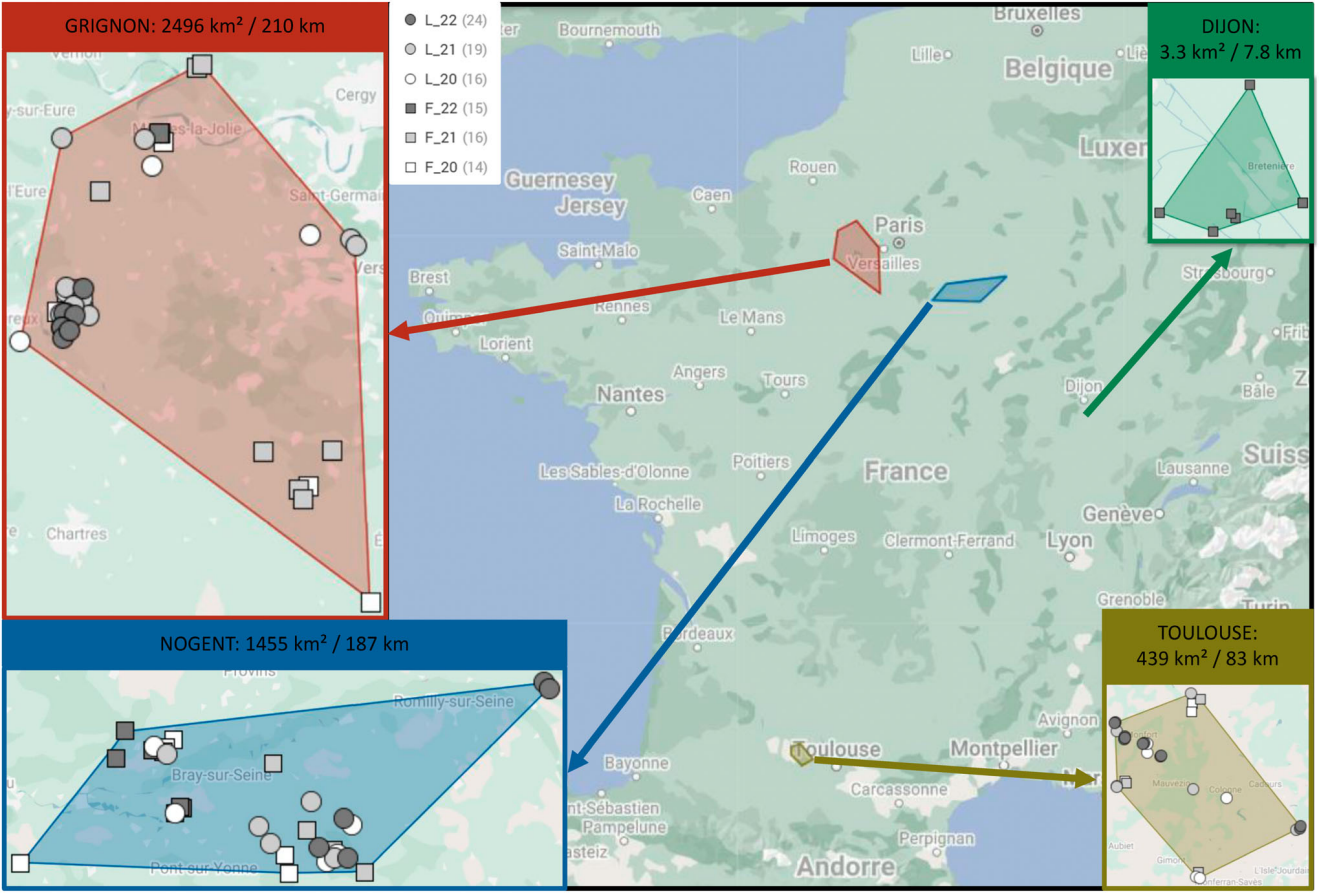
Location of the 59 lentil fields (L) and 45 faba bean fields (F) in the four French production basins (near Toulouse, Thiverval‐Grignon, Nogent‐sur‐Seine and Dijon) in 2020, 2021 and 2022. The coloured areas correspond to the extent of the study areas over the entire study period, with the surface area and perimeter indicated. Numbers following L and F codes refer to the year of survey, followed by the number of fields monitored [e.g. L_22 (24) stands for surveys conducted in 24 different lentil fields in 2022]

This study was conducted on French agricultural fields located in four production basins (near the cities of Toulouse, Thiverval‐Grignon, Nogent‐sur‐Seine and Dijon) over three consecutive growing seasons (2019–2020, 2020–2021 and 2021–2022), with the exception of the Dijon area, which was only surveyed in 2021–2022. In total, 59 lentil fields (16 in 2020, 19 in 2021 and 24 in 2022; Supporting Information Table S1) and 45 faba bean fields (14 in 2020, 16 in 2021 and 15 in 2022; Table S2) were monitored. However, not all fields were monitored and measured in exactly the same way, resulting in a different subset of fields being used for each analysis (Tables S1 and S2). These fields were managed either organically or without insecticide treatments (Fig. 1) to prevent the artificial suppression of bruchid populations and facilitate a more accurate observation of their dynamics. The average field size was 8.4 ± 5.0 ha for lentils (ranging from 0.7 to 23.7 ha; Table S1) and 7.6 ± 5.1 ha for faba beans (ranging from 0.9 to 23.4 ha; Table S2). The mean distance between fields of a given site and year was 15.4 ± 13.9 km for lentils (Table S3) and 22.0 ± 21.6 km for faba beans (Table S4).

Lentils were sown between 23 October and 27 April and harvested between 20 June and 5 August (Table S5), depending on the year and the production basin. Faba beans were sown between 20 October and 12 April and harvested between 15 June and 6 August (Table S6), depending on the year and the production basin. On average for all the fields, the rainfall from 1 January to 31 August was 384 mm (Tables S7 and S8). In terms of growing seasons, 2021 was the rainiest (459 mm), 2022 the driest (314 mm) and 2020 was intermediate (388 mm). In terms of sites, the cumulative rainfall was 435, 392, 341 and 331 mm for Nogent‐sur‐Seine, Grignon, Dijon and Toulouse, respectively. On average for all the fields, the cumulative degree‐days in base 10 °C (dd) during the same period were 1219 °dd (Tables S7 and S8). 2021 was the coldest season (970 dd), 2022 the warmest (1391 dd) and 2020 was intermediate (1284 dd). Regarding sites, the cumulative degree‐days were 1505, 1424, 1142 and 1020 dd for Toulouse, Dijon, Nogent‐sur‐Seine and Grignon, respectively.

### 2.2 Colonization and spatiotemporal distribution of adult bruchids

#### 2.2.1 Study design at field level

For each field, we made observations and took samples at various distances from the field edge, starting from the semi‐natural habitat (grass strip, hedge, wood) most represented on the perimeter. Samples were taken at various distances from the edge, with a particular focus on those taken close to it, in order to assess the gradual colonization of the plots from the edge. An observation area of 10 m long by 1.50 m wide was marked out along this edge. Parallel to it and in the direction of the centre of the field, four additional observation areas of identical dimensions were defined at 5, 15 and 30 m, as well as at 50 m in 2020 and in the field centre (≤390 and 610 m for faba beans and lentils, respectively; Tables S1 and S2) in 2021 and 2022.

#### 2.2.2 Sexing and identification of bruchids at the species level

Bruchid specimens were identified at the highest possible taxonomic level based on morphology. To achieve this, identification keys from the literature were used and the specimens compared with reference material from the Eco‐Entomology Laboratory collections.^12^, ^48^, ^49^, ^50^, ^51^ Sex identification is a prerequisite for species identification, because the criteria used to distinguish species, such as the configuration of the legs, the colour of the antennae, or the ornamentation of the elytra and pygidium, differ according to sex,^12^, ^48^, ^49^, ^50^, ^51^ In the genus *Bruchus*, males have slightly compressed, somewhat arched midtibiae bearing a lamella or spines at the apex, whereas females have simple midtibiae.^12^, ^48^, ^49^, ^50^, ^51^

#### 2.2.3 Monitoring of adult bruchids distribution by sweep net sampling

The spatiotemporal distribution of bruchids in the fields was monitored by sampling adults in each of the five observation areas at three phenological stages of the crop (vegetative, flowering and young pod corresponding to BBCH scale 30, 65 and 75, respectively).^52^ This was conducted on a total of 20 lentil fields (six in 2021 and 14 in 2022; Table S1) and 17 faba bean fields (four in 2021 and 13 in 2022; Table S2). The dates of sampling are indicated in Table S7 for lentil fields and Table S8 for faba bean fields. Samplings were carried out using a sweep net (diameter 50 cm, depth 90 cm; Cahurel Entomologie, Lopérec, France,), with 20 sweeps made in the crop over a length of 10 m. Collected bruchids were counted, sexed and identified to species level as described previously.

#### 2.2.4 Monitoring of crop colonization by adult bruchids using malaise traps

Bruchid colonization was monitored weekly in 2022 in five lentil fields (Toulouse area) and two faba bean fields (Dijon area) using unidirectional Malaise traps. We acknowledge the limited sample size of seven fields and a single year as a constraint. These results should be considered preliminary observations that require validation with broader sampling. The Malaise traps were placed in the middle of the observation area located along the field edge, positioned as close as possible to the first row of seedlings, and opened towards the outside of the field. The Malaise traps were installed at the vegetative stage of each crop: 29 March for lentils and 4 April for faba beans. Collection bottles were filled with a mixture of water, 1% colourless and odourless soap to reduce the water surface tension, and 50 g L^−1^ salt as a preservative.^53^ Samples were collected once a week for a period of 11 weeks (lentil) and 10 weeks (faba bean) after setting the traps. At each survey, the phenological stage of the crop was estimated using the BBCH scale. All bruchids collected were counted, sexed and identified to species level as described previously.

### 2.3 Estimation of bruchid‐damaged grains and identification of species

#### 2.3.1 Measurement of the percentage of bruchid‐damaged grains

At crop physiological maturity, 65 lentil pods and 25 faba bean pods were randomly collected from each of the four observation areas within the fields (5 m, 15 m, 30 m and >30 m), thus giving 200 lentil pods and 100 faba bean pods per field. This resulted in samples from 59 lentil fields (16 in 2020, 19 in 2021 and 24 in 2022; Table S1) and 45 faba bean fields (14 in 2020, 16 in 2021 and 15 in 2022; Table S2). The percentage of bruchid‐damaged grains–grains containing or having contained a developing bruchid–was then measured for lentil samples by crushing, and for faba bean samples by dissection, on 25 randomly chosen grains for each of the four observation areas within the fields, thus on 100 grains per field.

#### 2.3.2 Species and sex identification from emerged bruchid adults

At crop physiological maturity, 65 lentil pods and 25 faba bean pods were harvested along each of the four observation areas per field, thus 260 lentil pods and 100 faba bean pods per field. Sampling was conducted on randomly selected plants across 47 lentil fields (16 in 2020, 14 in 2021 and 17 in 2022; Table S1) and 42 faba bean fields (13 in 2020, 14 in 2021 and 15 in 2022; Table S2). The collected pods were placed in microperforated bottles and kept at 20 °C for 5 months to allow bruchids to emerge because some larvae remain in the grains for several months following a ‘late emergence’ phenological strategy.^9^, ^10^, ^24^, ^25^ The emerged bruchids were sexed and determined to species level as previously described, and counted. Seeds were then dissected to recover and to identify the nonemerged organisms.

### 2.4 Data analysis

All analyses were performed separately for faba bean and lentil. The fields included in each analysis are listed in Tables S1 and S2. Within each site, some fields were relatively close to each other (Tables S3 and S4) which may violate the assumption of independence between sites. However, the ‘Site’ and random ‘Field’ effects were included in the statistical models to account for this nonindependence.

In a first set of linear models, we compared the proportions of bruchid individuals and the proportion of females across species.

To analyze the temporal dynamics of bruchid colonization in Malaise traps, the total number of adults, females and males were, for each field, expressed as a percentage of the cumulative catches made during the entire monitoring period. We tested the ability of a Gompertz model based on cumulative dd with a base temperature of 10 °C to predict the colonization process for each bruchid species and both sexes.^47^ In our study, the sowing, first capture, and crop stage dates varied by field. For the latter, the temporal resolution of our observations did not allow us to precisely determine these dates. Therefore, we set the biofix date to 1 January, establishing a consistent reference point at which the Bruchids were in the same biological stage of dormancy. Under our climatic conditions, it takes a few months to accumulate more than 0 dd with a base temperature of 10°C. The Gompertz function A*exp(–B*exp(–C*dd)) was fitted to the data, where *A* is the load capacity parameter, *B* is a positive parameter that positions the origin of the transformed Gompertz model onto the vertical axis at time dd = 0, and *C* is a positive rate parameter. Parameter *A* was estimated as the average of the cumulative percentages reached in each of the five lentil fields or two faba bean fields by either the total catch or female or male catches. Parameters *B* and *C* were optimized by minimizing the root mean square error (RMSE) considering all observations (55 for lentil and 20 for faba bean). The models obtained were used to simulate the temporal dynamics of the female proportion within the bruchid population. Daily mean temperatures were obtained from the SAFRAN analysis system, which uses the optimal interpolation method to convert the climatic data provided by Météo‐France into an 8‐km grid.^54^ The data were downloaded via SICLIMA, a platform developed by AgroClim‐INRAE.

We then examined with linear models how the distance to the field edge and the crop stage influenced the spatial and temporal distribution of adult bruchids. The explanatory fixed‐effect of the distance variable was considered either as a numeric value corresponding to the distance from the field edge, or as a factor with four levels (5, 10, 30 and >30 m) or five levels (0, 5, 10, 30 and >30 m) in order to accommodate the uneven sample sizes across distances and to test for potentially nonlinear responses between distance classes.

Finally, we tested whether the percentage of bruchid‐damaged grains could be explained by bruchid population levels in the fields.

Measurements repeated within each field, at different crop stages and/or at different distances from the field edge were analyzed with generalized linear mixed models (lme4 package) with a random ‘Field’ effect, nested within each ‘Site’ whereas ‘Year’ could not be included in these models to avoid singularity owing to insufficient number of fields within some ‘Site’ × ‘Year’ combinations.^55^ *Post hoc* Tukey's honestly significant difference (HSD) tests were therefore performed when categorical fixed‐effect variables were significant (*P*‐value<0.05). Other data, first summarized across time and space for each field, were analysed with a generalized linear model (GLM, lme4 package) taking into account ‘Site’, corresponding to the production basin, and ‘Year’ as fixed‐effect cofactors. Furthermore, to identify the relative importance of site, field and distance in damage variability, we performed a partitioning of variance in seed damage between these three factors (varpart function of the vegan package).

All models assumed a negative binomial error distribution and a binomial error distribution for proportion data. Pseudo‐*R* ^2^ values were calculated with the *r.squaredGLMM* function (GLMM, generalized linear mixed model). The distribution of model residuals (normality, homoscedasticity, outliers) was checked and confirmed with the dharma package.^56^ All statistical analyses were performed with R software v3.1.3.^57^ Data analysis (explained variable, explanatory variables and models) altogether with statistical results are summarized in Table 1 for faba beans and Table 2 for lentils.

**Table 1:** Effects of bruchid species identity, individual sex, female abundance, distance to crop edge and crop stage on bruchid populations and/or on bruchid‐damaged grains in faba bean fields

Explained variable	Model	Figure	Explanatory variables	Estimate or estimated marginal means (±SE)	χ^2^	*P*‐value	Significant groups	*R* ^2^_m_	*R* ^2^_c_
Proportion of bruchid individuals by species	Binomial GLM	Fig. 2(A)	Species identity (five levels)		956	<10^−4^	***	0.97	
			*B. rufimanus*	0.88 ± 0.117			a		
			*B. signaticornis*	−1.97 ± 0.150			b		
			*B*. spp.	−2.03 ± 0.152			b		
			*B. rufipes*	−3.06 ± 0.223			c		
			*B. pisorum*	−4.62 ± 0.454			d		
Number of bruchids	Negative binomial GLM	Fig. 3(C)	Sex (male, ref = female)	0.04 ± 0.135	0.07	0.7889		0.44	
			Site (four levels)		23.49	<10^−4^	***		
			Year (three levels)		10.20	0.0061	**		
	Negative binomial GLMM	Fig. 5(A)–(C)	Stage (three levels)		64.53	<10^−4^	***	0.95	0.97
			*Vegetative*	−10.66 ± 35.95			ab		
			*Flowering*	−0.96 ± 0.53			a		
			*Young pod*	0.92 ± 0.49			b		
			Distance (five levels)	−0.01 ± 0.02	3.26	0.5160			
			Stage × Distance		12.91	0.1148			
		Fig. 5(G)–(I)	Stage (three levels)		69.98	<10^−4^	***	0.58	0.74
			*Vegetative*	−4.03 ± 0.93			a		
			*Flowering*	−0.65 ± 0.51			b		
			*Young pod*	0.97 ± 0.48			c		
			Distance (numeric)		1.13	0.2883			
			Stage × Distance		1.11	0.5754			
Percentage of bruchid‐damaged grains	Binomial GLMM	Fig. 6(A)	Distance (four levels)		6.16	0.1042		0.03	0.96
		Fig. 6(C)	Distance (numeric)	0.0003 ± 0.001	0.18	0.6751		0.00	0.96
		Fig. 7(A)	Number of females at young pod	0.01 ± 0.002	23.43	<10^−4^	***	0.47	0.47

Marginal (*R* ^2^_m_) and conditional (*R* ^2^_c_) coefficients of determination are shown for GLMM and only *R* ^2^_m_ for GLM. estimated marginal means are compared with *post hoc* Tukey's HSD tests for significant categorical variables. The asterisks indicate the statistical significance level (* for P‐value ≤ 0.05, ** for P‐value ≤ 0.01, and *** for P‐value ≤ 0.001).

**Table 2:** Effects of bruchid species identity, individual sex, female abundance, distance to crop edge, and crop stage on bruchid populations and/or on bruchid‐damaged grains in lentil fields

Explained variable	Model	Figure	Explanatory variables	Estimate or estimated marginal means (±SE)	χ^2^	*P*‐value	Significant groups	*R* ^2^_m_	*R* ^2^_c_
Proportion of bruchid individuals by species	Binomial GLM	Fig. 2(B)	Species identity (five levels)		17 884	<10^−4^	***	0.99	
			*B. signaticornis*	5.16 ± 0.28			a		
			*B*. spp.	−5.43 ± 0.30			b		
			*B. pisorum*	−7.11 ± 0.60			b		
			*B. rufimanus*	−8.21 ± 1.01			b		
			*B. griseomaculatus*	−8.21 ± 1.01			b		
Number of bruchids	Negative binomial GLM	Fig. 3(D)	Sex (male, ref = female)	0.006 ± 0.106	0.00	0.9537		0.25	
			Site (three levels)		27.65	<10^−4^	***		
			Year (Not included in the model to satisfy residual distribution)						
	Negative binomial GLMM	Fig. 5(D)–(F)	Stage (three levels)		26.47	<10^−4^	***	0.11	0.64
			*Vegetative*	2.58 ± 0.31			a		
			*Flowering*	2.06 ± 0.31			b		
			*Young pod*	1.86 ± 0.31			b		
			Distance (five levels)	−0.005 ± 0.002	2.93	0.5703			
			Stage × Distance		40.29	<10^−4^	***		
		Fig. 5(J)–(L)	Stage (three levels)		24.35	<10^−4^	***	0.06	0.56
			*Vegetative*	2.63 ± 0.32			a		
			*Flowering*	2.18 ± 0.32			b		
			*Young pod*	1.91 ± 0.32			b		
			Distance (numeric)		3.36	0.0670			
			Stage × Distance		3.56	0.1689			
Percentage of bruchid‐damaged grains	Binomial GLMM	Fig. 6(B)	Distance (four levels)		10.65	0.0138	*	0.01	0.98
		Fig. 6(D)	Distance (numeric)^a^	−0.001 ± 0.003	6.89	0.0087	**	0.03	0.98
		Fig. 7(B)	Number of females at young pod	0.002 ± 0.001	5.41	0.0199	*	0.12	0.46

Marginal (*R* ^2^_m_) and conditional (*R* ^2^_c_) coefficients of determination are shown for GLMM and only *R* ^2^_m_ for GLM. Estimated marginal means are compared with *post hoc* Tukey's HSD tests for significant categorical variables. The asterisks indicate the statistical significance level (* for P‐value ≤ 0.05, ** for P‐value ≤ 0.01, and *** for P‐value ≤ 0.001).

^a^ One outlier at 610 m was removed.

## 3 RESULTS

### 3.1 Diversity of bruchid species in fields and grains

**Figure 2:**
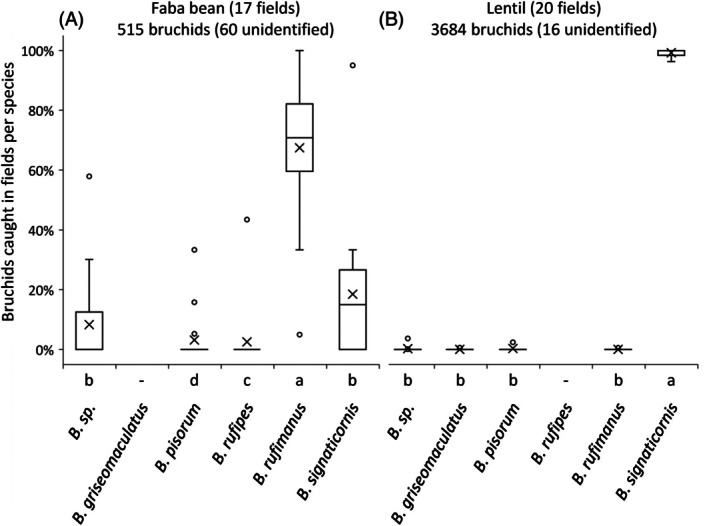
Bruchid species captured by sweep‐netting in faba bean (A) and lentil (B) fields in 2021 and 2022, considering all the sites. Individual data are expressed as a percentage of the sum, per field, of the individuals collected across three crop stages (vegetative, flowering and young pod) and four distances from the field edge (5, 15, 30 and >30 m). The crosses in the box plot correspond to the mean values

Considering all crop stages (vegetative, flowering and young pods) and all four observation areas within each field (5, 15, 30 and >30 m), sweep net sampling enabled the identification of four bruchid species in faba beans [Fig. 2(A)] and four bruchid species in lentils [Fig. 2(B)]. Among the 455 bruchids identified in faba beans, a significantly (Table 1, *post hoc* Tukey's HSD test) higher proportion were *B. rufimanus* (74.5%), compared to *B. signaticornis* (19.8%) and two other marginal species (*B. pisorum* and *B. rufipes*). The 3668 bruchids identified in lentils were almost exclusively *B. signaticornis* (99.6%; Table 2), with a sporadic presence of *B. griseomaculatus*, *B. pisorum* and *B. rufimanus* [Fig. 2(B)]. Only 1.8% of the bruchids could not be identified because of damage during capture or transport (60 of 515 for faba bean, and 16 of 3684 for lentil).

**Figure 3:**
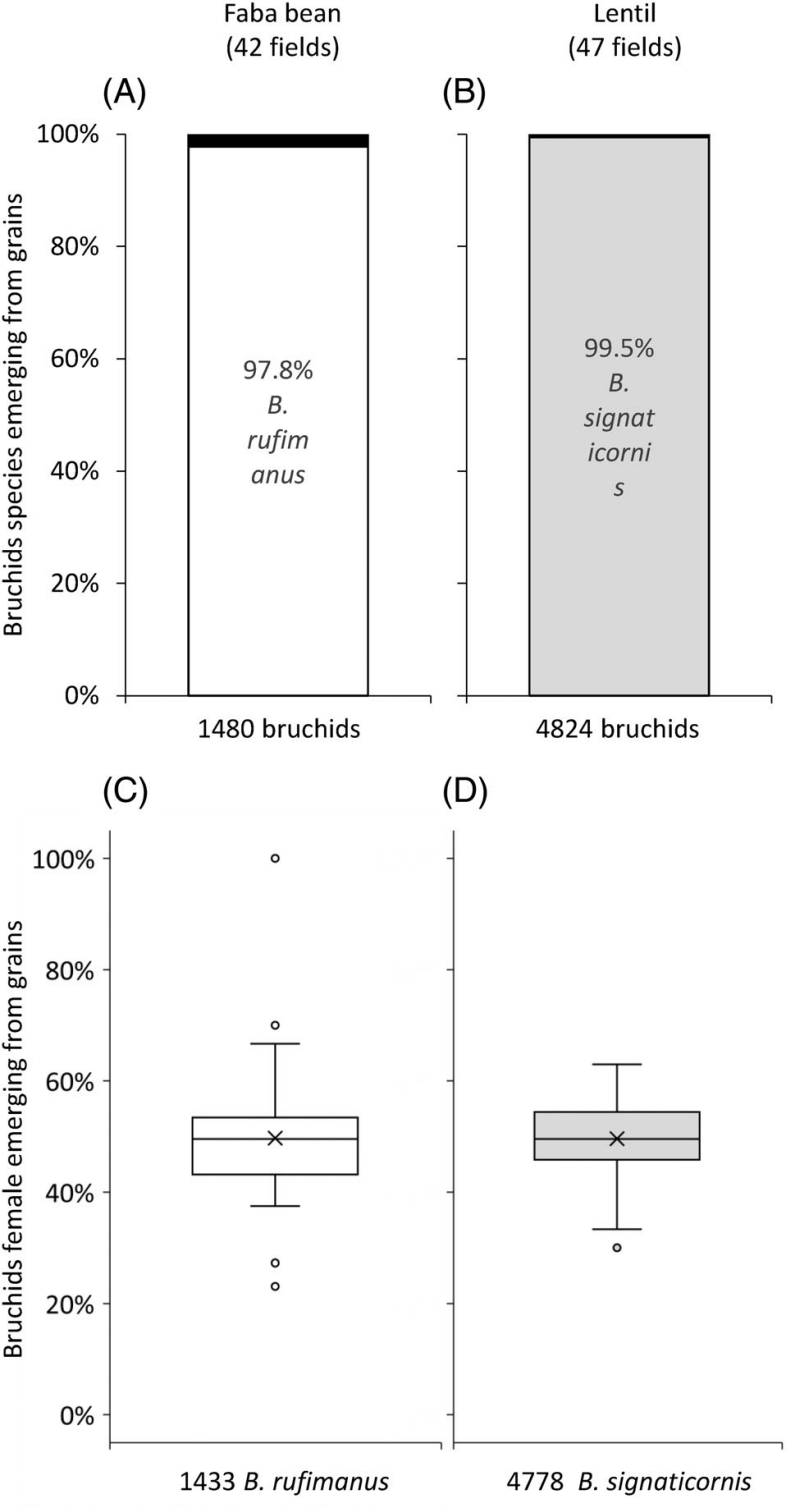
Species composition (A, B) and proportion of females (C, D) of bruchids emerging from faba beans (A, C) and lentils (B, D) grains harvested in 2020, 2021 and 2022, considering all sites. Individual data are expressed as a percentage of the sum, per field, of the individuals collected at four distances from the field edge (5, 15, 30 and >30 m). The crosses in the box plot correspond to mean value

Of the bruchids emerging from grains [Fig. 3(A), (B)], 97.8% belonged to *B. rufimanus* for faba bean (*n* = 1443 of 1480 emergences) and 99.5% to *B. signaticornis* for lentil (*n* = 4798 of 4824 emergences). The remaining individuals could not be identified to species level because they were fragments of adults, larvae or pupae. Among the bruchid individuals identified, the proportion of females [Fig. 3(C), (D)] was 49.7 ± 12.3% for *B. rufimanus* in faba bean and 49.6 ± 6.8% for *B. signaticornis* in lentil, which did not significantly differ from a 1:1 female–male ratio for both faba bean (*P* = 0.79; Table 1) and lentil (*P* = 0.95; Table 2).

### 3.2 Temporal dynamics of bruchid colonization in fields

**Figure 4:**
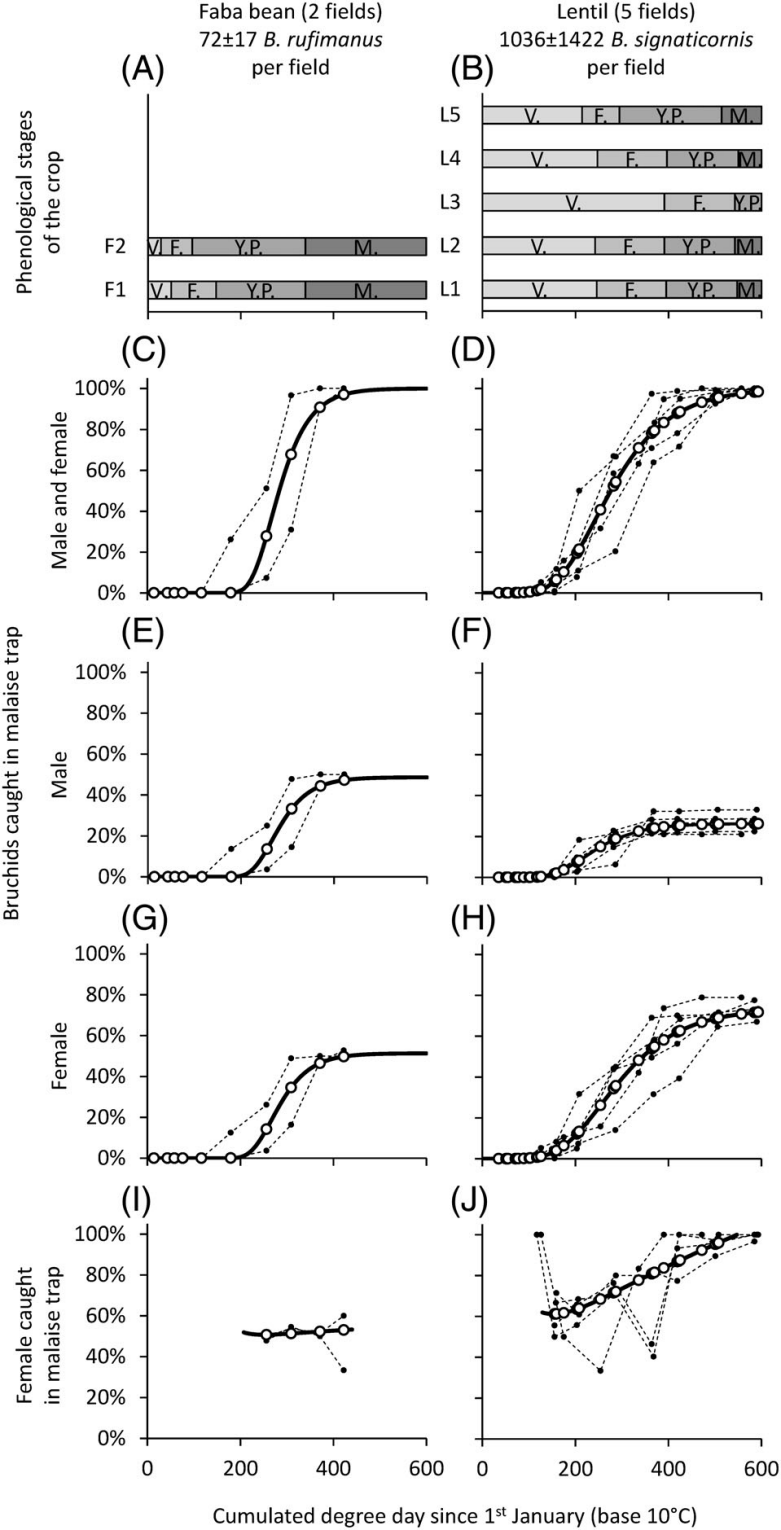
Phenological stages of the crop (V, vegetative; F, flowering; YP, young pods; M, maturity; (A, B), cumulative bruchid captures when considering all individuals (C, D), males only (E, F), and females only (G, H) and proportion of females (I, J). Individual data are expressed as a percentage of the total number of individuals caught per field in the Malaise trap in 2022 over the entire period considering only *B. rufimanus* in faba beans (A, C, E, G, I) and only *B. signaticornis* in lentils (B, D, F, H, J). The data are expressed as a function of cumulative dd with a base temperature of 10 °C and a biofix date set at 1 January. Closed symbols connected by a dotted line correspond to observed values, whereas open symbols connected by a solid line correspond to fitted values according to the Gompertz function. These fitted values were subsequently used to estimate the temporal dynamics of the proportion of females

Colonization of faba bean fields by *B. rufimanus* (Fig. 4), as detected by the Malaise traps, began between 179 and 256 dd, corresponding to the young pod stage [Fig. 4(A), (C)]. Colonization by *B. signaticornis* in lentil fields occurred between 89 and 159 dd, during the vegetative stage [Fig. 4(B), (D)]. A Gompertz model based on cumulative dd provided a good fit to the observed colonization dynamics confirming that a base temperature of 10 °C together with a biofix set at 1 January enable the arrival of bruchids in the fields to be predicted correctly. Note that parameter *A* was estimated for the total catch, female catches and male catches as 100%, 73.6% and 26.4% for lentils, and 100%, 51.4% and 48.6% for faba beans, respectively. The cumulative number of captured individuals increased from 5% to 95% over a period of 181 dd in faba beans with a peak colonization rate of 0.83% per dd at 267 dd [Supporting information Fig. S1(A)]. For lentils, the cumulative number of captured individuals increased from 5% to 95% over a period of 346 dd with a peak colonization rate of 0.43% per dd at 245 dd [Fig. S1(B)].

In faba bean fields, colonization by *B. rufimanus* was synchronous between sexes [Fig. 4(I)], with females representing 51% of individuals captured throughout the monitoring period. Colonisation of lentil fields by *B. signaticornis* showed a marked sex imbalance [Fig. 4(J)], with females accounting for 74% of captures over the entire period. Moreover, *B. signaticornis* males tended to arrive at the beginning of colonization. Thus, 62% of the *B. signaticornis* males were captured between 0 and 287 dd [corresponding to the vegetative stage or the onset of flowering in lentils; Fig. 4(B)], whereas 56% of the *B. signaticornis* females were captured between 287 and 593 dd. This temporal offset led to a progressive increase in the proportion of *B. signaticornis* females, from 67% over the period 0–287 dd compared to 80% over the period 287–593 dd [Fig. 4(J)]. Notably, at 287 dd, 49% of all *B. signaticornis* individuals (males and females combined) were captured in lentil fields, a value that closely matches the 50% population predicted by the Gompertz model (276 dd).

### 3.3 Spatial and temporal distribution of bruchids and damage

**Figure 5:**
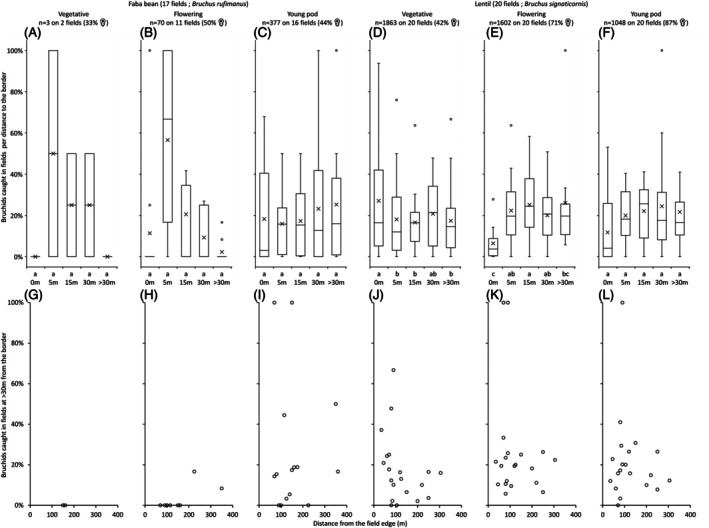
*Bruchus rufimanus* in faba bean (A–C, G–I) and *B. signaticornis* in lentil (D–F, J–L) captured by sweep netting in 2021 and 2022, considering all sites, across three phenological stages (vegetative, flowering, and young pod) as a function of distance from the field edge. (A–F) Considers five distance classes (0, 5, 15, 30 and >30 m) whereas (G–L) considers the continuous gradient of distances beyond 30 m from the field edge (Fig. S2 for partial residuals). The crosses in the box plot correspond to mean value. Individual data are expressed as a percentage of the total number of individuals caught per field across the five distances at a given phenological stage

In faba bean, the number of *B. rufimanus* adults captured with the sweep net increased significantly over time across phenological stages (vegetative, flowering and young pod) (*P* < 0.0001; Table 1), but remained low with mean counts per observation area (all distances combined) of 0.04 ± 0.18 individual at vegetative stage, 0.82 ± 2.01 at flowering and 4.44 ± 7.78 at young pod stage [Fig. 5(A)–(C)]. In the faba bean fields, the proportion of female *B. rufimanus* appeared to be relatively stable over time with 33%, 50% and 44% at the vegetative, flowering and young pod stages, respectively [Fig. 5(A)–(C)]. *B signaticornis* adults captured with the sweep net in lentils were significantly higher at the vegetative stage compared to the flowering and young pod stages (*P* < 0.0001; Table 2). Mean numbers of *B. signaticornis* adults caught per observation area were 18.63 ± 23.21, 16.02 ± 27.67 and 10.48 ± 17.27 at vegetative, flowering and young pod stages, respectively [Fig. 5(D)–(F)]. In the lentil fields, the proportion of female *B. signaticornis* increased over time with 42%, 71% and 87% at the vegetative, flowering and young pod stages, respectively [Fig. 5(D)–(F)].

At all three phenological stages of the crop, when the five distance classes were compared, there was no significant effect of distance from the field edge on the number of *B. rufimanus* adults caught in faba bean fields [*P* = 0.52; Table 1; Fig. 5(A)–(C)]. For *B. signaticornis* in lentil fields [Fig. 5(D)–(F)], distance had a stage‐dependent effect (*P* = < 10–4; Table 2). At the vegetative stage, significantly more *B. signaticornis* individuals were captured at the field edge (0 m) than further inside the field. However, at flowering and young pod stages, the number of *B. signaticornis* adults caught at 0 m decreased, becoming lower than at the inner distances (5, 15, 30 and >30 m).

Taking into account the continuous gradient of distances beyond 30 m from the field edge, the percentage of bruchids captured was not significantly correlated with distance for either faba bean [*P* = 0.29; Table 1; Figs 5(G)–(I) and S2(A), (C), (E) for partial residuals] or lentil [*P* = 0.07; Table 2; Figs 5(J)–(L) and S2(B)–(F) for partial residuals].

**Figure 6:**
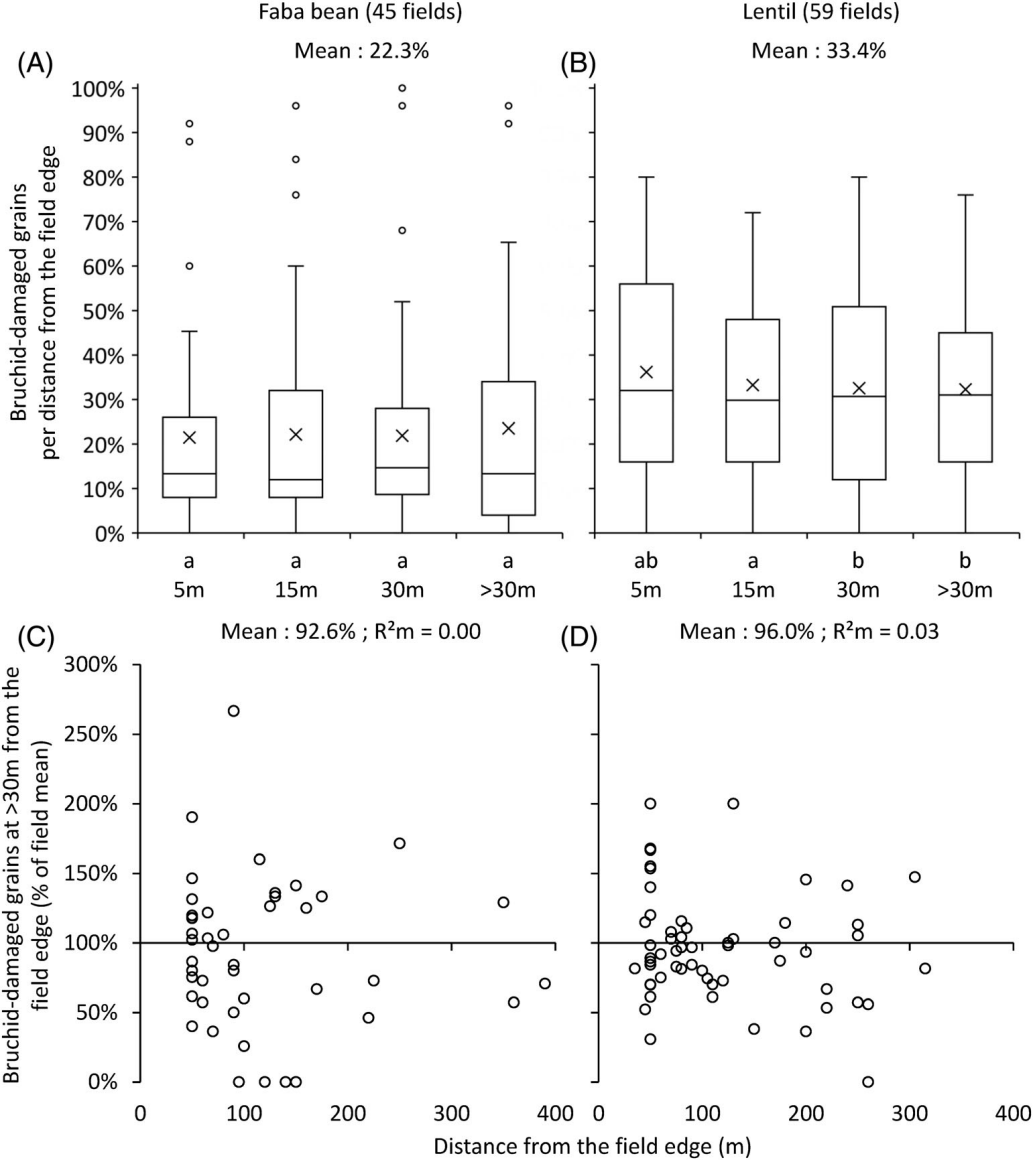
Bruchid‐damaged grains, considering all years and sites, in faba bean (A, C) and lentil (B, D) as a function of distance from the field edge. (A, B) considers four distance classes (5, 15, 30 and >30 m;) whereas (C, D) consider the continuous gradient of distances beyond 30 m from the field edge (Fig. S3 for partial residuals). The crosses in the box plot correspond to mean value. Individual data are expressed, or as a percentage of the total number of grains per field in each distance class (A, B), or as a percentage of the average damage of the field (C, D). One outlier at 610 m was removed (B, D)

The median proportion of bruchid‐damaged grains was 13.3% (interquartile range: 8.0 to 29.0%) for faba beans [Fig. 6(A)] and 30.3% (interquartile range: 16.0–48.1%) for lentils [Fig. 6(B)]. The effects of distance from the field edge on bruchid‐damaged grains were similar to those on bruchid populations. In faba bean [Fig. 6(A)], there were no significant differences in the percentage of bruchid‐damaged grains among distance classes [*P* = 0.10; Table 1). In lentils [Fig. 6(B)], bruchid‐damaged grains decreased slightly with distance from the field edge (*P* = 0.01; Table 2). Over the entire continuous gradient of distances beyond 30 m from the field edge [Fig. 6(C), (D)], no significant relationship was found between the percentage of bruchid‐damaged grains and distance in faba beans [*P* = 0.68; Table 1 and Fig. S3(A) for partial residuals]. In lentil fields, a slight decrease was observed with distance [*P* = 0.0087; Table 2 and Fig. S3(B) for partial residuals]. The partitioning of variance indicated that 69% and 70% of the total variance in seed damage was attributed to ‘Field identity’, for faba bean and lentil, respectively, whereas ‘Distance’, alone and in interaction with other factors, was related to <1% of total variance in both crops.

### 3.4 Relationship between the percentage of bruchid‐damaged grains and the number of females captured at the young pod stage

**Figure 7:**
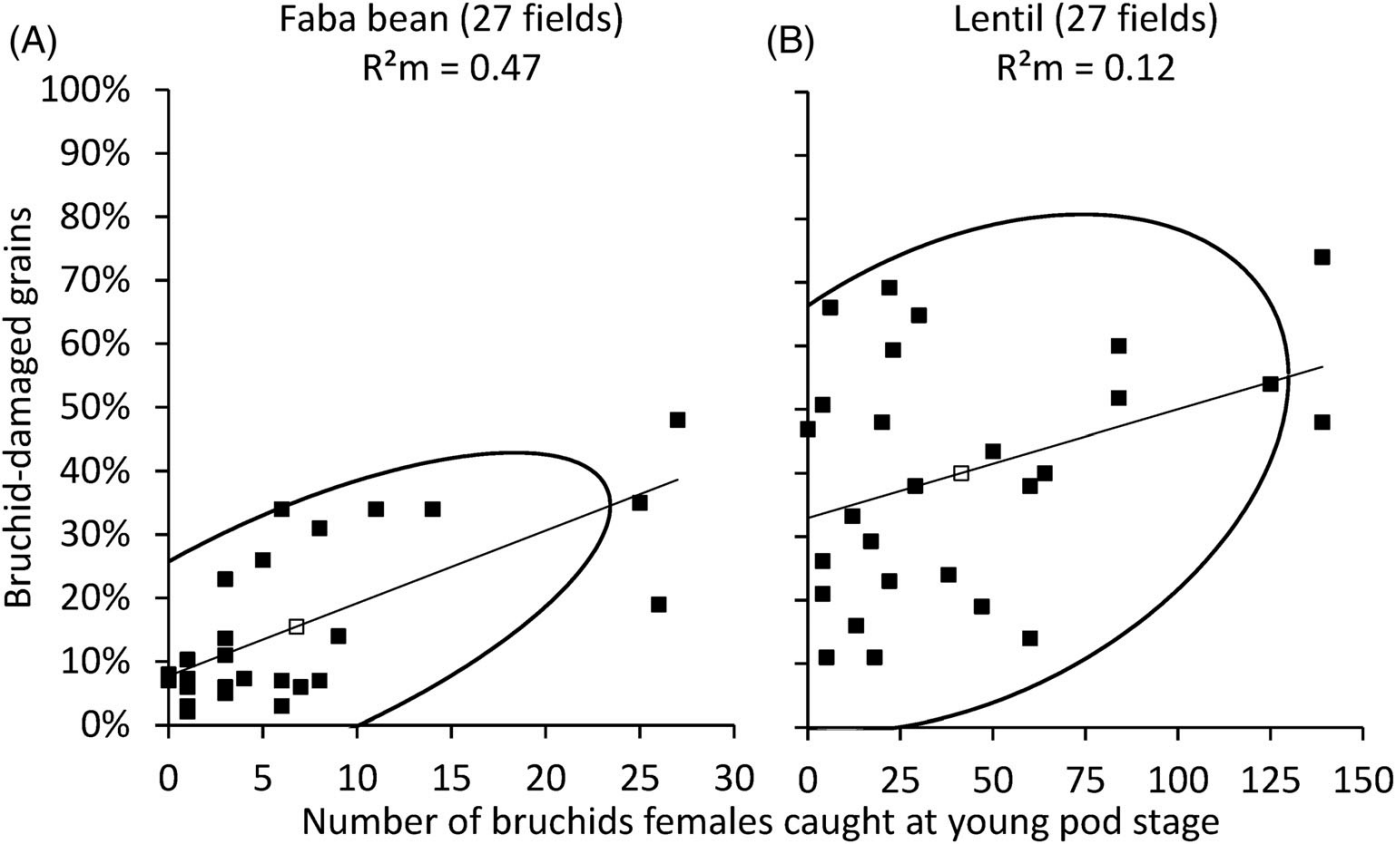
Relationship between bruchid‐damaged grains and the number of bruchids female captured at the young pod stage in 2021 and 2022, considering all sites, for *B. rufimanus* in faba bean (A) and *B. signaticornis* in lentil (B). The number of females corresponds to the total number captured at young pod stage across four distances from the field edge (5, 15, 30 and >30 m). The bruchid‐damaged is expressed as a percentage of the total number of grains per field across the four distances. Solid lines represent the linear regression between the two variables. Ellipses represent the 90% prediction interval. The open square corresponds to the overall mean

There was a positive and significant relationship between the average percentage of bruchid‐damaged grains and the number of female bruchids captured at the young pod stage, which corresponds to the oviposition period, for both faba beans [*P* < 0.0001; Table 1; Fig. 7(A)] and lentils [*P* = 0.0199; Table 2; Fig. 7(B)]. This general trend was observed across all data collected in the field over the 3 years, after averaging the abundance of bruchids and damage per field across the four distances. Data dispersion was high in both cases. For *B. rufimanus* on faba beans, the y‐intercept was 7.7% and the relationship had a slope of 1.1% meaning that a 10% increase of bruchid‐damaged grains was done by nine females. For *B. signaticornis* on lentils, the y‐intercept was 32.9% and the slope 0.2% indicating that a 10% increase of bruchid‐damaged grains was done by 59 females.

## 4 DISCUSSION

### 4.1 One pest species per crop and opportunistic species

Previous studies on bruchids often failed to distinguish clearly between larval feeding within grains from adult feeding on plants.^12^, ^13^ Also, recent literature suggests that many earlier works contain species identification errors and may therefore overestimate the total number of species capable of successfully completing their larval development on faba bean and lentil.^12^, ^13^, ^14^, ^15^, ^16^, ^17^, ^18^, ^58^, ^59^, ^60^ To date, 13 species have been identified as capable of completing their development on lentils, and seven on faba beans.^14^, ^15^, ^16^, ^17^, ^18^ Among them, recent literature considers *B. signaticornis* and *B. rufimanus* to be the primary pest species of lentil and faba bean, respectively, in France and Europe.^9^, ^10^, ^11^ The results obtained in our study, conducted in four different regions over 3 years, confirm these recent findings by identifying *B. signaticornis* and *B. rufimanus* as the only bruchid pest species causing damage on lentils and faba beans in France, respectively.

Adults of *B. pisorum*, *B. rufipes* and *B. signaticornis* were occasionally recorded on faba beans, whereas *B. griseomaculatus*, *B. pisorum* and *B. rufimanus* were recorded on lentils. However, *B. signaticornis* on faba beans and *B. pisorum* on lentils were not capable of completing their development on these crops.^14^, ^15^, ^16^, ^17^, ^18^ Considering the differences between species in the number of adults captured in the fields and the absence of any species other than *B. rufimanus* on faba bean and *B. signaticornis* on lentil emerging from harvested grains, the most likely hypothesis is the opportunistic presence of adults of *B. pisorum*, *B. rufipes* and *B. signaticornis* on faba bean, as well as *B. pisorum*, *B. griseomaculatus* and *B. rufimanus* on lentil. Indeed, adult bruchids are capable of feeding on a variety of cultivated and spontaneous Fabaceae plants. Therefore, it is hypothesized that females may select other nearby host plants for oviposition, such as *Pisum sativum*, identified as a preferred host of *B. pisorum* and a frequent crop in the study areas.^61^ The same hypothesis is formulated regarding *B. griseomaculatus*, which has the potential to successfully complete its larval development in lentil grains, but which is generally found in low abundance and for which lentil may serve only as an occasional host.^17^

Likewise, the presence of *B. signaticornis* in faba bean fields, before the colonization of lentil fields, indicates the availability of nutritional resources sought and exploited by this bruchid. Indeed, faba beans produce extrafloral nectar, which has been identified as a potential food source for adult bruchids, along with floral nectar and pollen.^62^ Observations made during our field surveys confirmed that extrafloral nectar was available on faba beans and could have been exploited by adults of *B. signaticornis* before the onset of lentil flowering. Nonetheless, the present study did not observe *B. signaticornis* adults feeding directly on extrafloral nectar on faba beans. Further research is therefore required to validate this hypothesis. In any case, the presence of *B. signaticornis* in faba bean fields suggests new possibilities for monitoring and controlling this pest. In particular, a ‘pull’ strategy would require, on the one hand, the identification of the specific factors responsible for attracting *B. signaticornis* to faba bean (e.g. semiochemical, visual or nutritional) and, on the other, a thorough assessment of the risk that faba bean could act as a temporary reservoir, enabling the survival and persistence of adult bruchids until lentil crops reach a phenological stage suitable for oviposition and larval development.^63^

### 4.2 Activity periods and temporal dynamics of female proportions for *B. signaticornis* and *B. rufimanus*

Bruchid samples taken at key phenological stages of the crops within fields provided results consistent with the monitoring data obtained from Malaise traps. From a methodological point of view, the latter observations should be considered with caution because of the sample size limited to seven fields and to a single year. Additionally, the capture efficiency of Malaise traps could have been influenced by their positioning, particularly in relation to nearby semi‐natural habitats, and prevailing winds that could affect both bruchid flight and Malaise trap performance.^11^ Despite efforts to standardize trap location and orientation, we cannot be certain that trapping conditions were consistent across fields or that they accurately reflected actual colonization dynamics. Therefore, these results should be considered preliminary observations requiring broader sampling with greater spatial and temporal coverage to confirm our findings regarding the colonisation patterns of bruchid pests.

For *B. rufimanus* on faba beans, traps recorded first individuals between 179 and 256 dd (base 10 °C), which corresponded to the young pod stage. Conversely, catches with sweep nets revealed first individuals being caught at the vegetative stage and increasing significantly over time across phenological stages (vegetative, flowering and young pod). Catches at the vegetative and flowering stages therefore certainly anticipated the mass arrival of bruchid in the field. Therefore, it would have been relevant to continue net sampling beyond the young pod stage in order to measure the peak of the adult population abundance, which, according to the Gompertz model based on cumulative degree‐days, occurred at around 267 dd–thus, shortly after the arrival of the first individuals. These observations are in line with previous observations in Algeria, France, Great Britain, Sweden or Latvia mentioning a population peak between full flowering and the onset of pod formation.^11^, ^19^, ^21^, ^22^, ^24^, ^25^ Adult activity, concentrated over a period of approximately 2 weeks under our conditions and synchronized for both sexes, suggests a potential single wave of colonization that could be predicted using the Gompertz model based on cumulative dd, opening up the possibility of optimizing chemical treatments.

For lentil bruchids, the first bruchids arrived between 89 and 159 dd (base 10 °C) and the estimated population peak made with the Malaise traps at ≈245 dd, which corresponds broadly with the beginning of flowering, aligning closely with the maximum number of adults captured in fields during the vegetative stage. For *B. signaticornis* on lentil fields, colonization occurred over a period of approximately 1 month, from the vegetative to the flowering stages. Our results showed a concomitant arrival of males and females at the vegetative stage, which corroborates previous observations made in France.^10^ However, a higher proportion of males than females has been observed at the beginning of the lentil growth cycle, whereas our results indicate a predominance of females, including during the period from 0 to 287 dd (corresponding to the vegetative stage or the flowering onset).^10^ Notably, we observed that female proportion increased over time, a trend consistent with previous research and consistent between samples taken at key phenological stages of the crops within fields and between the monitoring data obtained from Malaise traps.^10^ The prolonged activity of *B. signaticornis* females in lentil fields suggests the availability of sufficient food resources for maintenance, pod exploration and oviposition. This may be facilitated by lentil's indeterminate flowering period, which can even be prolonged by favourable soil and climatic conditions. Additionally, the presence of nutritional resources in the surrounding landscape such as neighbouring crops (e.g. oilseed rape) and wild plants (e.g. *Prunus* spp. and *Crataegus* spp.), may support extended adult activity.^19^

### 4.3 Uniform distribution across the fields

Several authors suggested that flying bruchids have a long‐distance dispersal capacity, but this has never been quantified with precision.^11^, ^15^, ^49^ Only one work on *B. pisorum*, reported the capacity of the pea bruchid to fly >20 m high in cultivated areas and to overwinter ≤5 km away from pea crops.^45^ Despite this lack of information, it is reasonable to assume that bruchids are capable of long‐distance movement, given their small size and flight ability, an analogy with comparable species such as *Psylliodes chrysocephala* or *Brassicogethes aeneus*, for which quantitative data exist.^64^, ^65^ Our results seem consistent with rapid movement of the bruchids in the crop, resulting in a relatively homogeneous spatial distribution of adults in fields with an average area of 8.4 ha for lentils and 7.6 ha for faba beans, at the vegetative stage for *B. signaticornis* and at the flowering‐young pod stage for *B. rufimanus*. For lentil fields in particular, the significantly negative effect of distance was very small and limited to the vegetative and flowering stages of bruchid colonization. It would have been relevant to anticipate the first captures by sweep‐netting to better characterize the spatiotemporal process of field colonization from the edges. More generally, a better understanding of bruchid dispersal capacities would enhance our understanding about their landscape distribution, between overwintering sites and crops, and would open up avenues for biological control.

### 4.4 A partial relationship between the number of bruchids and the percentage of bruchid‐damaged grains

In view of the precocious reproductive maturity in female bruchids of *B. signaticornis* and *B. rufimanus*, it is known that mating in *B. signaticornis* typically occurs at the beginning of field colonization, after which the males may die.^10^, ^19^, ^66^ The number of females captured at the young pod stage, which corresponds to the oviposition period, can serve as an indicator of the potential pressure exerted by pests on the crop in order to predict the percentage of bruchid‐damaged grains. Although the relationship between these two variables was statistically significant, it showed a high degree of dispersion. This could be due to insufficient sampling, as suggested by the high percentage of damaged grains (>30%) observed in lentil crops, even though very few adult bruchids were captured [Fig. 7(B)].

Another plausible explanation is that the quantity of pods and seeds available for egg laying by the same number of females could vary from one field to another, depending on the varieties grown, crop development conditions and agricultural practices. This source of variation was not taken into account in the design of our study. A single female *B. signaticornis* can lay eggs on ≈100 pods.^67^ This requires intense movement around the field to locate sufficiently unoccupied pods, only if females are receptive to the presence of traces associated with conspecific egg‐laying. Therefore, for the same number of females, a greater quantity of available pods or higher number of grains per pod, both variables not measured in this study, would mechanically reduce the percentage of bruchid‐damaged grains. This could partly explain the discrepancy between the number of *B. signaticornis* adults at the young pod stage (higher at 0 m than at the inner distances) and the percentage of bruchid‐damaged grains (which decreases slightly with distance from the field edge).

However, the wide dispersion of the data could also suggest that regulatory factors were modulating the percentage of bruchid‐damaged grains. Mortality during embryonic (egg stage) and larval development could vary according to climatic conditions or plant varietal effect.^20^, ^24^ For example, rain can remove eggs from pods, whereas high temperatures can lead to egg desiccation.^9^, ^23^ The ability of 1^st^‐instar larvae to penetrate into the pod probably depends on the toughness of the pod wall.^23^ Natural enemies, such as predators and oophagous parasitoids, also could reduce the percentage of bruchid‐damaged grains but never act uniformly from one field to another, from one region to another, or from one year to another. Although bruchid parasitoids seem to prefer attacking larval stages, this has been demonstrated in *B. signaticornis* on lentils.^10^ Further work therefore seems necessary to quantify the effects of these factors in order to better predict the percentage of bruchid‐damaged grains and thus develop effective methods to reduce crop damage.

## 5 CONCLUSION AND PERSPECTIVES

Management methods for bruchids, other than chemical control, are currently under evaluation and development.^9^, ^68^ Their successful implementation requires in‐depth knowledge of the ecology of bruchids and their interactions with their environment.^69^ This study, conducted across four French regions over three growing seasons, focused on lentil and faba bean bruchids. Its first objective was to consolidate recent findings and clarify the ecology of species infesting these crops in the French context. In particular, we have confirmed that *B. rufimanus* and *B. signaticornis* are, at least in France, the only species damaging faba beans and lentils, respectively. We also have demonstrated that the spatial distribution of bruchids and grain damage are uniformly distributed according to distance from the field edge, indicating strong dispersal capabilities. Furthermore, the proportion of bruchid‐damaged grains was high for both crops, with considerable variability, which confirms that bruchids, by limiting crop productivity and contributing to unstable yields, pose a considerable challenge to the wider adoption of these crops by farmers.

Additionally, the high degree of dispersion in the relation between female abundance and bruchid‐damaged grains highlights the importance of regulatory factors influencing larval and egg survival. A detailed analysis of the processes that ultimately determine the percentage of bruchid‐damaged grains is therefore needed, taking into account the dynamics and distribution of female egg‐laying, egg viability, and the ability of larvae to penetrate pods and grains. This knowledge is crucial to ultimately contribute to the development of alternative control solutions to insecticides that target different ecological functions and different development stages. Likewise, the occurrence of *B. signaticornis* in faba bean fields, where the larvae are unable to complete their development, suggests new possibilities for monitoring and controlling this pest in lentil fields. This can be achieved through the implementation of a ‘pull’ strategy, with faba bean serving as a trap crop.^63^

Finally, an analysis of the effects of functional biodiversity (e.g. natural enemies) at different scales (within the field, local environment and the surrounding landscape), in interaction with climatic conditions, local practices and landscape structure, is required to improve our understanding of the dynamics of bruchid populations, the variability of damage and the natural regulation by parasitoids. The integration of these scientific insights could facilitate the design of comprehensive pest management strategies aimed at mitigating the adverse effects of bruchids and reducing the use of chemicals.

## FUNDING INFORMATION

This study was funded by a research project involving InVivo Group (ex‐Soufflet Group) and the National Research Institute for Agriculture, Food and the Environment (INRAE). Y.T. received additional support from the SPECIFICS (ANR‐20‐PCPA‐0008) project funded by the ‘Growing and Protecting crops Differently’ French Priority Research Program (PPR‐CPA), as part of the national investment plan operated by the French National Research Agency (ANR). C.H. was funded by a postdoctoral grant from the AgroEcoSystem department of INRAE.

## CONFLICT OF INTEREST

The authors declare no competing interests.

## AUTHOR CONTRIBUTIONS

A.C.‐L., A.G., Y.T., M.G., A.‐S.V. and L.B. designed the study and conducted the research; A.C.‐L. and L.B. wrote the first draft; All co‐authors revised the paper.

## Supporting information


**Figure S1.** Bruchid capture rate (% of individuals caught per dd) in the Malaise trap in 2022 calculated from fitted values according to the Gompertz function. The data are expressed as a function of cumulative degree‐days with a base temperature of 10 °C and a biofix date set at 1 January. Here, we considered only *B. rufimanus* in faba beans [two fields; Fig. S1(A), (C), (E)] and *B. signaticornis* in lentils [five fields; Fig. S1(B), (D), (F)].

**Figure S2.** Partial residuals for the numbers of *B. rufimanus* on faba bean (17 fields) and *B. signaticornis* on lentil (20 fields) at the vegetative, flowering and young pod stages as a function of distance from the field edge for all sites and years. Grey shading indicates a 95% confidence interval around the regression line.

**Figure S3.** Partial residuals for the percentage of bruchid‐damaged grains on faba beans (45 fields) and lentil (59 fields) as a function of distance from the field edge for all sites and years. Grey shading indicates a 95% confidence interval around the regression line.

**Table S1.** List of lentil fields used for the different figures according to year (20 = 2020 etc.) and site (N, Nogent‐sur‐Seine; T, Toulouse; G, Thiverval‐Grignon; D, Dijon) with the mean distance to the other fields of the same site and year (‘Distance’), field surface (‘Size’) and distance from field edge to centre (‘Centre’).

**Table S2.** List of faba bean fields used for the different figures according to year and site (see Table S1 for details) with the mean distance to the other fields of the same site and year (‘Distance’), field surface (‘Size’) and distance from field edge to centre (‘Centre’).

**Table S3.** Distance between lentil fields of the same year and site (see Table S1 for details).

**Table S4.** Distance between faba bean fields of the same year and site (see Table S1 for details).

**Table S5.** Dates of sowing, harvest and sampling of the lentil fields according to year and site (see Table S1 for details).

**Table S6.** Dates of sowing, harvest and sampling of the faba bean fields according to year and site (see Table S1 for details).

**Table S7.** Cumulated precipitation and cumulated dd (base 0°C and base 10°C) of the lentil fields according to year and site (see Table S1 for details).

**Table S8.** Cumulated precipitation and cumulated dd (base 0 °C and base 10 °C) of the faba bean fields according to year and site (see Table S1 for details).

## Data Availability

The data that support the findings of this study are available from the corresponding author upon reasonable request.
