# Supplementary figures and images for: Colonization and spatiotemporal distribution of bruchid pests in lentil and faba bean fields
Source: Pest Manag Sci. 2026 Feb 23;82(6):5780–94. doi: 10.1002/ps.70679 (PMC13158440; doi:10.1002/ps.70679)

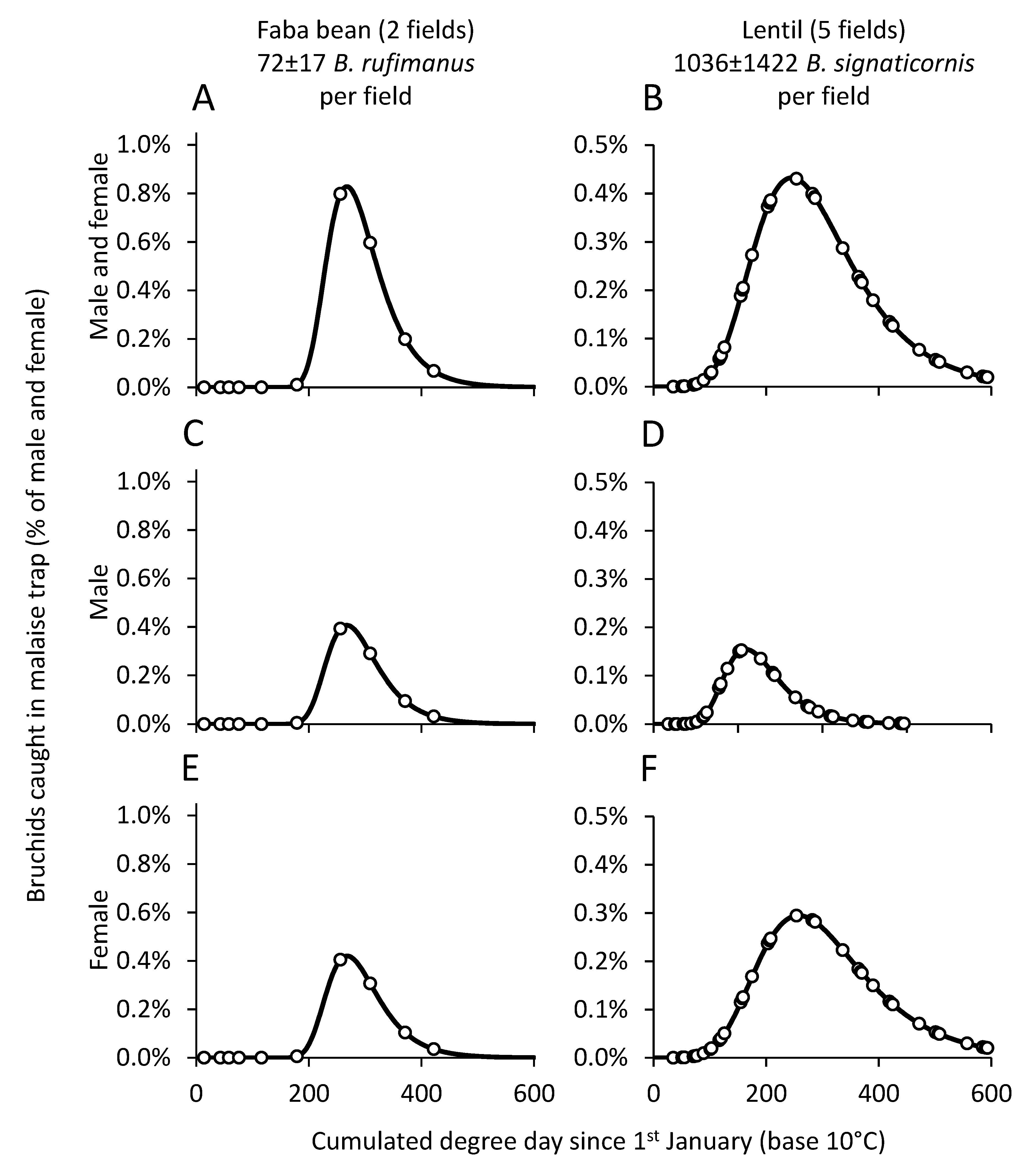

Supplement: Supplementary file 1 — Figure S1. Bruchid capture rate (% of individuals caught per dd) in the Malaise trap in 2022 calculated from fitted values according to the Gompertz function. The data are expressed as a function of cumulative degree‐days with a base temperature of 10 °C and a biofix date set at 1 January. Here, we considered only B. rufimanus in faba beans [two fields; Fig. S1(A), (C), (E)] and B. signaticornis in lentils [five fields; Fig. S1(B), (D), (F)]. [file PS-82-5780-s011.png]

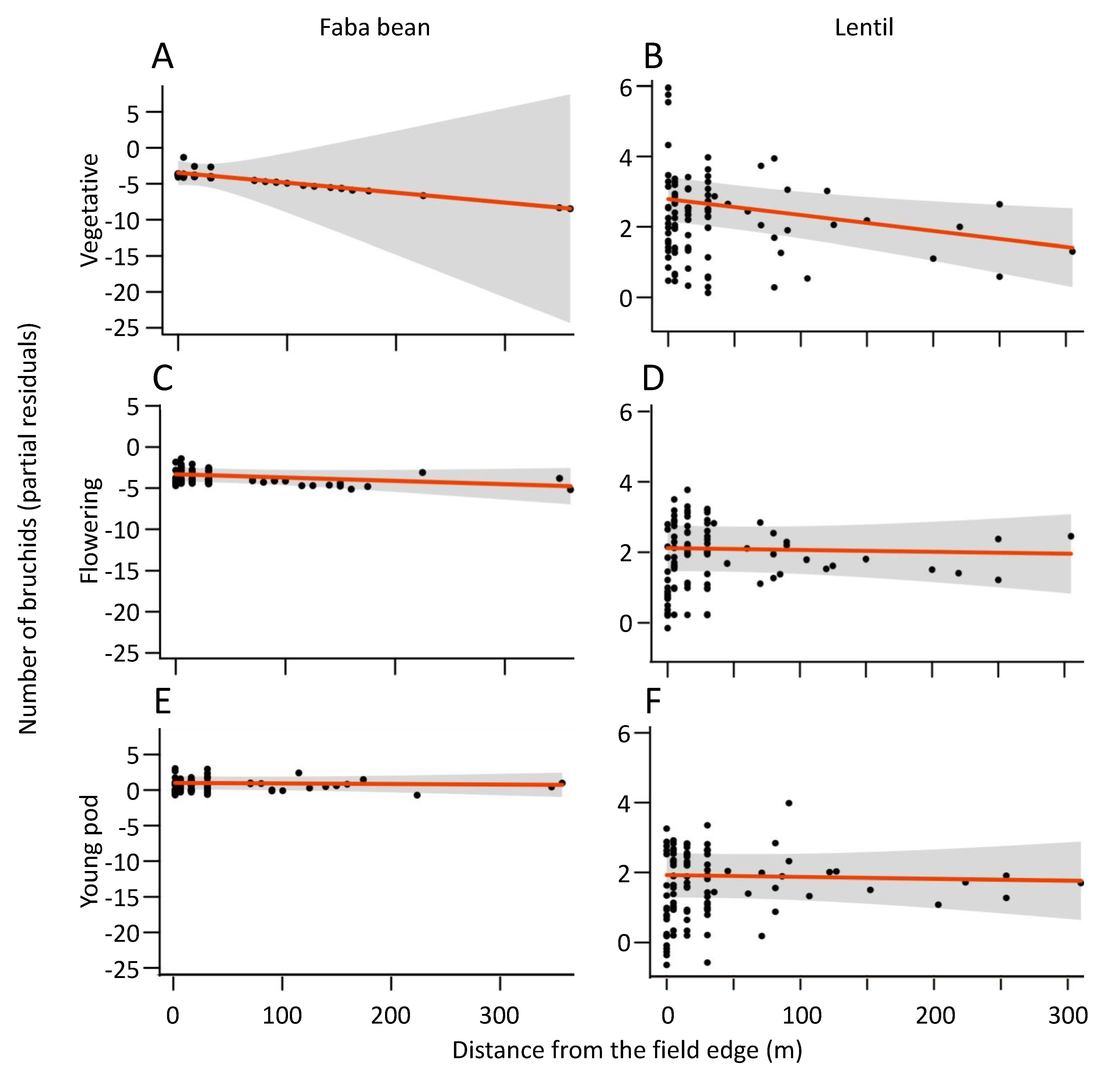

Supplement: Supplementary file 2 — Figure S2. Partial residuals for the numbers of B. rufimanus on faba bean (17 fields) and B. signaticornis on lentil (20 fields) at the vegetative, flowering and young pod stages as a function of distance from the field edge for all sites and years. Grey shading indicates a 95% confidence interval around the regression line. [file PS-82-5780-s003.png]

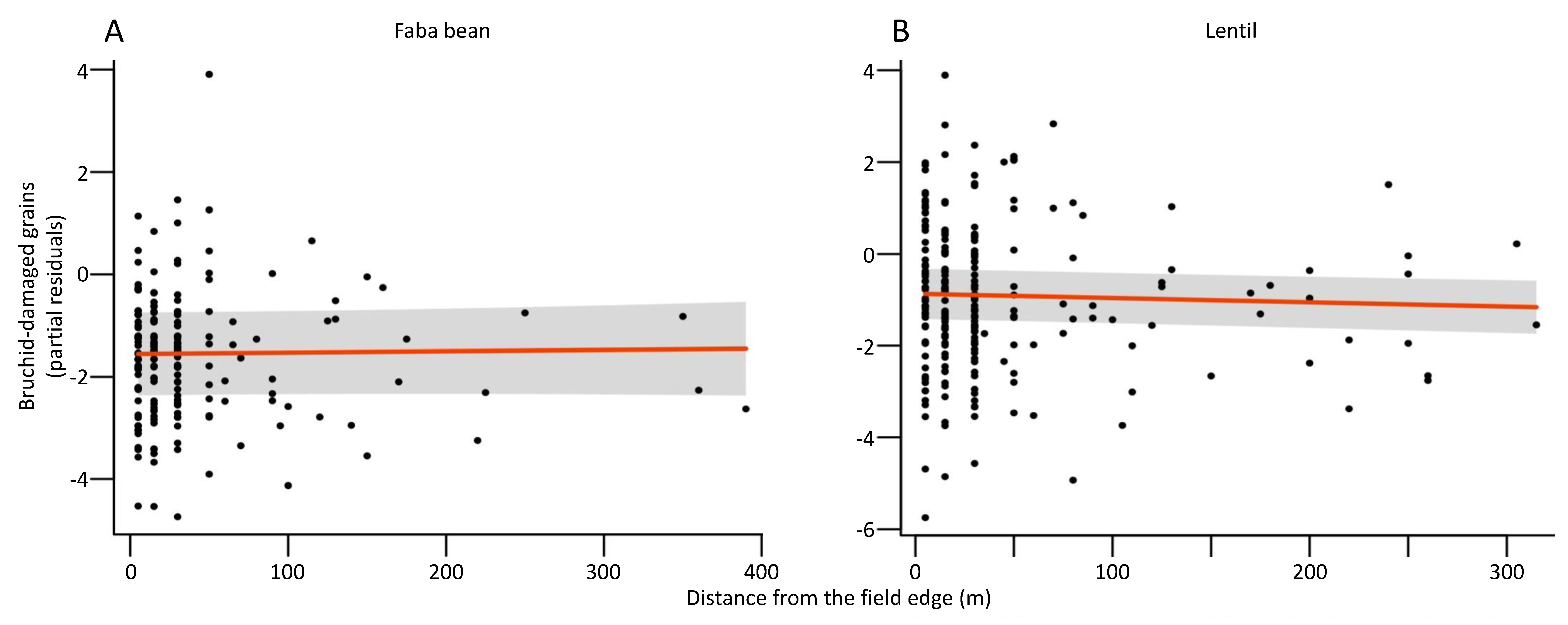

Supplement: Supplementary file 3 — Figure S3. Partial residuals for the percentage of bruchid‐damaged grains on faba beans (45 fields) and lentil (59 fields) as a function of distance from the field edge for all sites and years. Grey shading indicates a 95% confidence interval around the regression line. [file PS-82-5780-s001.png]
